# Polyphenolic drug composition based on benzenepolycarboxylic acids (BP-C3) increases life span and inhibits spontaneous tumorigenesis in female SHR mice

**DOI:** 10.18632/aging.101024

**Published:** 2016-08-28

**Authors:** Vladimir N. Anisimov, Irina G. Popovich, Mark A. Zabezhinski, Maria N. Yurova, Margarita L. Tyndyk, Ivan V. Anikin, Peter A. Egormin, Irina A. Baldueva, Elena I. Fedoros, Sergey E. Pigarev, Andrey V. Panchenko

**Affiliations:** ^1^ N.N. Petrov Research Institute of Oncology, Pesochny-2, Saint-Petersburg 197758, Russia; ^2^ RD Pharm Ltd., Saint-Petersburg 192012, Russi

**Keywords:** polyphenolic compound BP-C3, biomarkers of aging, spontaneous tumorigenesis, mice

## Abstract

Effects of long-term application of novel polyphenolic composition BP-C3, containing polyphenolic benzenepolycarboxylic acids, vitamins and minerals on some biomarkers of aging, life span and spontaneous tumorigenesis has been studied in female SHR mice. Administration of BP-C3 with drinking water (0.005%) did not exert any toxic effect (did not have effect on general condition of animals, weight dynamics and consumption of food), postponed age-related switch-off of estrous function, caused slight reduction of body temperature. An increased survival was observed in mice treated with BP-C3 (p=0.00164, log rank test). BP-C3 increased mean lifespan – by 8.4%, lifespan of the last 10% of animals – by 12.4%, and life span of tumor-free mice – by 11.6%. A tendency in ability of BP-C3 to inhibit development of spontaneous tumors in mice was detected, though it did not reach the level of statistical significance (p=0.166, log rank test). The number of malignant mammary tumors was 1.5 times less and total number of tumors of various localizations was 1.6 times less in BP-C3 treated animals. Multiple tumors were registered in 8% of mice in the control group and no cases – in BP-C3 treated group. Thus, BP-C3 demonstrated some anti-carcinogenic and a pronounced geroprotective activity.

## INTRODUCTION

BP-C3 is a composition jointly developed by N.N. Petrov Research Institute of Oncology and Nobel Ltd. [1] and comprising a novel polymer BP-Cx-1 (produced under the license from RD Innovation ApS, Denmark), iron complex, selenium, ascorbic acid and retinol. Currently, the composition is going through preclinical testing as a supportive care drug for oncology patients treated with chemotherapeutics.

BP-Cx-1 polymer, comprising benzenepolycarboxylic acids (polyphenolic compounds), produced from lignin and resembling structure of plant flavonoids, is used as a carrier-ligand in a novel anti-tumor platinum analogue BP-C1 [2] and in a novel radio-mitigator – BP-C2, containing molybdenum complex [3].

Selenium and iron are introduced into the composition, to correct for possible deficit of microelements that may arise from long-term consumption of benzene-polycarboxylic acids that exert chelating effects. Low concentrations of selenium in blood plasma (< 40-60 μg/l) are associated with an increased risk of development of lung, colon and prostate cancers [4-6]. Uptake of iron, that belongs to the group of essential microelements, is reported to be considerably reduced when consuming food containing polyphenols: catechins, flavonols, flavones, anthocyanins, pro-anthocyanidins and phenolic acids. Ascorbic acid is reported to be able to eliminate this negative effect of polyphenolic compounds [7]. For these reasons, along with iron the composition was supplemented with ascorbic acid.

Recent studies indicate that two known antioxidants retinol (25 – 350 mg per day) and ascorbic acid (200 – 1000 mg per day) in doses used in the clinical practice do not exert positive effects on either longevity or risk of development of cancer [8]; nor do they improve outcomes of the main anticancer therapy [9]; doses above clinical cause some adverse events [10].

On the other hand, scientific literature contains evidence suggesting retinoids to be able to reduce toxicity of a number of anti-tumor agents as well as to improve efficacy of interferon-alpha-2a in treatment of T-cell lymphoma and cervical cancer [11, 12]. Authors also believe that these are not the only cancer indications (and localizations) where retinoids can produce favorable effects.

In accordance with this, to correct for vitamin deficiency, unavoidable in cancer patients undergoing chemotherapy, BP-C3 composition was supplemented with low doses of ascorbic acid and retinol.

The study was performed to assess safety of BP-C3 as a candidate compound for supportive care in cancer patients undergoing chemotherapy, and effect of long-term administration (life-time) of this compound on longevity and tumorigenesis in laboratory mice was evaluated.

## RESULTS

### Age-related body weight dynamics

**Figure 1:**
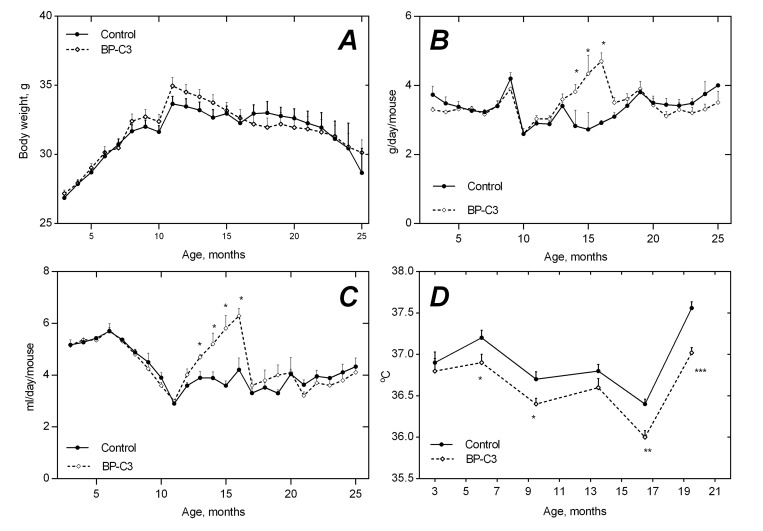
Age-related dynamics of body weight (**A**), food consumption (**B**), water consumption (**C**) and body temperature (**D**) in SHR mice non-treated and treated with BP-C3. * - The difference with control at the same age is significant, p<0.05.

The body weight of mice in both groups (control and BP-C3 group) increased with age, exceeding at 11 months the body weight of 3-month-old mice by 25.3% in the control group, and by 28.7% in the group treated with BP-C3. Beyond the age of 11 months, the body weight of mice in both groups was stabilized and gradually decreased towards the age of 2 years. Differences in the body weight of the control and the BP-C3 group animals were not statistically significant (Fig. 1A).

### Age-related dynamics of food and water consumption

The rather synchronous changes in food (3-5 g/mice/day) and water (3-6 ml/mice/day) consumption were observed in both groups at different time points and correlated with each other to a certain extent (Fig. 1B and Fig. 1C). BP-C3 did not have significant effect on patterns of water and food consumption as compared with the control. An exception was registered at the age of 14-16 months, where a significantly higher food and water consumption (p<0.05) was observed in the BP-C3 group.

### Age-related dynamics of body temperature

Gradual decrease of the body temperature was observed in mice of both groups between the age of 3 and 17 months followed by a sharp rise afterwards. A slight, but statistically significant reduction of the body temperature in mice given BP-C3 in comparison with the control group was registered at the age of 6, 9.5, 16.5 and 19.5 months (Fig. 1D). Lowest body temperature in both groups was registered at the age of 16.5 months: 36.4±0.06°C in the control group and 36.0±0.08°C in the BP-C3 group, p<0.01.

### Age-related dynamics of estrous function in mice

**Figure 2:**
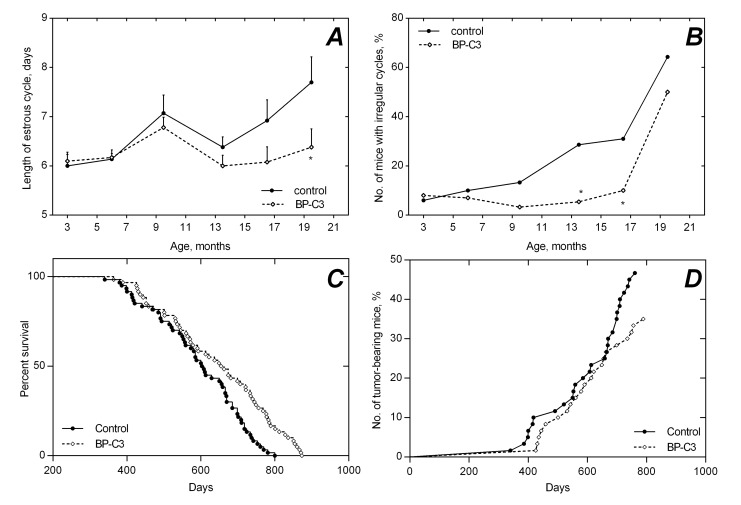
Age-related dynamics of the length of estrous cycles (**A**), fraction of mice with regular estrous cycles (**B**), survival (**C**) and tumor yield (D) in SHR mice non-treated and treated with BP-C3. * - The difference with control at the same age is significant, p<0.05.

Disturbances of estrous function, such as elongation of estrous cycles and decrease of incidence of mice with regular estrous cycles, were observed in the control group starting from the age of 13.5 months (Fig. 2B). The length of estrous cycles was increased in the control females after the age of 13.5 months, and at the age of 19.5 months it was 1.4 days longer than in mice treated with BP-C3: 7.7±0.5 days vs. 6.4±0.4 days, correspondingly (p<0.05). These changes were not observed in the BP-C3 group until the age of 19.5 months. The differences in the fraction of mice with regular cycles between the control and the BP-C3 treated mice became significant at the age of 13.5 and 16.5 months (p<0.05).

### Survival and longevity of female SHR mice

Survival dynamics in the control and in the BP-C3 groups are presented in Table 1. There is significant similarity between the rate of survivors in both groups until the age of 600 days. However, to the age of 700 days survived 23.3% of control mice and 41.6 % of mice given BP-C3 (p<0.05) and to the age of 800 days – 1 out of 60 control mice and 10 out of 60 mice treated with BP-C3 (p<0.01). The survival curve for BP-C3 group was shifted to the right, as compared with that for control mice.

**Table 1:** Effect of treatment with BP-C3 on survival of female SHR mice

Group	Number of survivors at the age of: (days)							
	100	200	300	400	500	600	700	800	900
Control	60	60	60	56	45	32	14	1	0
BP-C3	60	60	60	58	49	35	25*	10*	0

* p<0,05

The Log-rank (Mantel-Cox) test confirmed the highly significant difference in survival distribution between the control and the BP-C3 groups (p=0.0015). The contribution of the last quartile had the major impact (p<0.0001). No differences in survival in the first quartile of the deceased animals were observed (p=0.088).

As it can be seen from the data presented in Table 2, mice from the BP-C3 group lived, in average, 50 days longer than mice in the control group (8.4%, p<0.05). Age of the last mouse that died in the BP-C3 group was 873 days, whereas in control group it died at the age of 800 days (+9.1%, p<0.01). The mean life span of the last 10% survivors in the BP-C3 group was 3 months longer (+12.4%, p<0.01) than that of the last decile in the control group.

**Table 2:** Effect of treatment with BP-C3 on survival of female SHR mice

Parameters	Control	BP-C3	Δ,%; p
Number of mice	60	60	
Mean life span, days (M±m)	596 ± 15,5	646 ± 18,7	+8.4%; p<0.01
Maximum life span, days	800	873	+9.1%
Mean life span of 10% last survivors, days	768 ± 8,5	863 ± 3,7	+12.4%; p<0.01
Number of tumour-free mice	31 (52%)	39 (65%)	+13%; p=0.19
Mean life span of tumour-free mice, days	606 ± 21,7	676 ± 24,3	+11.6%; p<0.05

### Development of spontaneous tumors and non-tumor pathologies in female SHR mice

The dynamics of age-related increase in spontaneous tumor development is presented on the Figure 2D. The first tumor in the control group was detected at the age of 291 days, while the first tumor in the BP-C3 group was detected at the age of 338 days (1.2 months later). The Log-rank (Mantel-Cox) test showed non-significant difference in distribution of tumors development between the control and the BP-C3 groups (p=0.166). Mean life span of tumor-bearing mice in both groups was similar, however the mean life span of tumor-free mice was significantly longer in animals treated with BP-C3 (p<0.05; Table 3).

**Table 3:** Effect of treatment with BP-C3 on incidence of spontaneous tumors and non-tumor pathology in female SHR mice

Parameters	Control	BP-C3
Number of mice	60	60
Total number of tumor-bearing mice^2^	29 (48%)	21 (36%)
Number of malignant tumor-bearing mice^2^	27 (45%)	20 (34%)
Number of tumors	34	21
Number of malignant tumors	32	20
Number of tumors per tumor-bearing mice	1,17 ± 0,05	1,00 ± 0,00*
Age at the first tumor detection, days	291	338
Mean life span of tumor-bearing mice, days	595 ± 24,2	592 ± 25,3
*Localization and type of tumors (number of cases, %)*		
Mammary adenocarcinoma	22 (33%)	15 (25%)
Malignant lymphoma	6 (10%)	4 (7%)
Lung adenoma	1 (2%)	1 (2%)
Lung adenocarcinoma	1 (2%)	1 (2%)
Subcutaneous fibrosarcoma	2 (3%)	-
Cavernous liver hemangioma	1 (2%)	-
Ovarian granulesa cell tumour	1 (2%)	-
Non-tumor pathology (number of cases, %)		
Pneumonia	25 (42%)	30 (50%)
Hepatitis	5 (8%)	3 (5%)
Nephropathy	5 (8%)	8 (13%)
Enteritis	3 (5%)	5 (8%)
Adnexitis	3 (5%)	2 (3%)
Peritonitis	2 (3%)	0
Bleeding	0	1 (2%)

* *–* p<0,05

Tumors were detected in 29 out of 60 mice in the control group (48%) and in 21 out of 60 mice given BP-C3 (36%) (p=0.19). In total, 34 tumors were discovered in 29 tumor-bearing control mice. It is worth noting that multiple tumors were observed in 8% of control mice: two mice had 2 mammary adenocarcinomas each and 3 animals in this group had a combination of mammary tumors and malignant lymphoma, or a combination of mammary tumors and malignant lymphoma. There were no cases of multiple tumors in the group of mice treated with BP-C3 (p=0.057).

Mammary tumors, mostly solitary, were the predominant type of tumors developed in both groups (Table 3). These tumors were morphologically classifi-ed as type A mammary adenocarcinomas, and some of them expressed invasion into the surrounding tissues [13]. Malignant lymphomas were the second most frequent type of tumors detected in both groups. The majority of them were malignant disseminated lymphomas with lesions to all peripheral lymph nodes and enlarged liver and para-aortic lymph nodes. In two mice of the control group with malignant lymphoma the thymus was primarily involved into malignant process. Lung neoplasms were detected in 2 animals from the control group and in 2 animals of the BP-C3 group. In both groups, these neoplasms, located beneath pleura, had a form of grey-pink 0.1 – 0.2 cm nodes or single larger nodes, reaching 0.5 cm in size. At histological examination, these small nodes presented themselves as trabecular or tubular adenomas, composed of uniform oval cells, supposedly of alveolo-genic origin, and were surrounded by a capsule. Larger tumor nodes, that were detected in one animal from the control and one animal from the BP-C3 group, were classified as papillary adenocarcinomas. Some solitary cases of tumors of other types were detected in the control group (Table 3). Frequency of non-tumor pathologies did not differ statistically between the groups (Table 3).

## DISCUSSION

In this paper for the first time it was shown that novel polyphenolic composition based on benzenepoly-carboxylic acids (BP-C3) increases lifespan and alleviates spontaneous tumor development. Long-term administration of 0.005% solution of BP-C3 with drinking water to female SHR mice does not induce toxic or any other serious adverse effects. The compound does not exert any significant effect on body weight, food and water consumption, thus further confirming the safety.

One of the important findings of the study was decrease of body temperature throughout lifetime of mice treated with BP-C3 as compared with the control animals. Transgenic mice with a reduced by 0.5°C core temperature are reported to live longer than wild type mice [14]. In mice treated with BP-C3 we observed the decrease in body temperature in 0.2-0.4°C range. Long-term reduction of body temperature and related slowdown of metabolic processes are known to increase lifespan of animals exposed to calorie restriction, to melatonin or dipeptide Lys-Glu [15-17]. It is worth mentioning that these compounds are shown to exhibit stronger or weaker immunomodulatory properties.

The parameters of function of reproductive system could serve as non-invasive and adequate indicators of biological age of animals [18]. One of them is the length of estrous (ovulatory) cycle, which increases with the age in mice and rats. Another, much more important parameter, is the age-related dynamics of estrous cycles. In rodents with spontaneous ovulation, e.g. in mice and rats of the majority of strains, the estrous function usually discontinues at the age of 13-17 months, followed by periods of persistent estrous and anestrous being equivalent to climacteric and menopause. With the age, fraction of mice with regular estrous cycles gradually decreases. In our study, comparative analysis of estrous function, performed in both groups of animals, clearly demonstrated ability of BP-C3 to slow-down aging of the reproductive system. This was manifested both by a relatively higher frequency of regular cycles in animals in the age of 13.5-19.5 months and by a slow-down of the age related extension of estrous cycles in animals treated with BP-C3 (Fig. 2A, B).

Some other compounds and drugs with geroprotective activity are also shown to postpone age-related switching-off of the estrous function in mice and rats: melatonin, polypeptide pineal preparation Epitalamin, synthetic tetrapeptide Ala-Glu-Asp-Gly, neuro-protective delta-sleep inducing peptide (DSIP, Trp-Ala-Gly-Gly-Asp-Ala-Ser-Gly-Glu), antidiabetic bigunanides buformin, phenformin and metformin, mitochondria-targeted plastoquinon-containing anti-oxidant drug SkQ1 and some other [19-24]. Pharmacologically active compounds of different classes, with different biological efficacy and different specific mechanisms of action in relation to aging, can be considered as anti-aging drugs. These drugs are potent antioxidants.

As demonstrated in our study, tumor-free animals treated with BP-C3 had a significantly longer (by 2.3 month) lifespan as compared with the control. Structure of non-tumor pathology was similar in both control and treated with BP-C3 mice. The fact that in animals exposed to BP-C3 unfavorable outcomes of diseases were considerably postponed, as compared to untreated animals, may indicate an ability of this compound to inhibit immuno-aging.

Age-related deterioration of immune system functions may result in reduced ability of monocytes and dendritic cells to recognize its native (tumor) or foreign (viral) antigens and lowered immunogenic representation of antigens by T-lymphocytes; lower number of naive T- and NK-cells and increased number of T-lymphocytes with depleted functional capabilities [25, 26]. T. Fulop et al. [27] demonstrated that such changes are tightly associated with misbalance in subpopulations of T-lymphocytes and low probability of immune response to new antigens. As it has been established, functional activity of T-cells and macrophages that reduces with age, bringing down antiviral, antibacterial and anti-tumor defense of the body, is directly associated with considerably reduced production of IL-2 [28].

As it was shown, aging processes in C57Bl/6 and Balb/c mice are also associated with reduction of functional activity of macrophages (inclusive of the pro-inflammatory IL-6, TNF-α and IL-1β), which has a negative effect on ability of the immune system to resist infections [29-31]. Besides, some cytokines (TNF-α and INFγ) have been shown to be efficient in inhibiting exponential growth of tumor cells, aiding in their elimination [32].

In earlier studies [33] looking at immunomodulatory effects of BP-C3 it has been demonstrated *in vitro* that polyphenolic component of BP-Cx-1 promotes activation of monocytes and production of a range of cytokines (IL-1β, IL-6, IL-25, GM-CSF, INFγ, TNFα). Similar results were reported for immunomodulatory compound Vilon® (synthetic thymic peptide), which also exerts immunomodulatory efficacy: in-vitro Vilon® increased expression of receptors on T- and B-lymphocytes in patients with secondary immuno-deficiencies and induced production of IL-1α, IL-1β, IL-8 and TNF-α [34].

Thus, there are reasons to suggest that immuno-modulatory properties of BP-C3 can have effect on aging of the immune system of animals and be responsible for its anti-aging effect. A comparison of observed effects of BP-C3 and effects reported for a number of other polyphenolic compounds supports a hypothesis of similarity of their capacity to extend life span. Thus, polyphenolic compound, curcumin, was able to increase mean life span of rats and C57Bl/6 mice [35, 36]. Green tea polyphenols also exhibited anti-ageing effect in mice of this strain [37], and resveratrol could extend median lifespan of C57Bl mice on high-calorie diet [38].

In our study, for the first time, it was shown that BP-C3 – structural analogue of flavonoids, alongside with increasing the life span, also inhibited spontaneous tumorigenesis. In mice treated with BP-C3, first tumors appeared 1.5 months later than in the control group. We found rather strong tendency to decrease of the incidence and multiplicity of tumors in mice treated with BP-C3. These data suggest cancer preventing potential of BP-C3. As a number of other gero-protectors, BP-C3 has a true anti-carcinogenic effect. The suppressive effect of geroprotectors on carcinogenesis was demonstrated in a number of models where it was mainly realized through normalization of hormonal-metabolic and immunological parameters, oxidative stress and control of proliferative and inflammatory status [23, 24, 39-43].

It should be mentioned specifically that BP-C3 belongs to a class of polyphenolic compounds, anti-ageing effects of which are still poorly understood. BP-C3 belongs to the family of compounds based on the novel BP-Cx-1 ligand derived from lignin. This family includes several compounds exhibiting radio-protective, radio-mitigating and antitumor effects. Investigation of molecular mechanisms of action of BP-C3 could be one of the most promising directions of further research.

## MATERIALS AND METHODS

### Animals

Outbred female SHR mice were purchased from “Rappolovo” (Russia) animal nursery. Mice were kept 5-7 in polypropylene cages (30 × 21 × 10 cm) under standard 24-hour light-dark regimen (12 hours light: 12 hours darkness) at 22±2 ˚C, and received standard laboratory chow from Laboratorkorm Ltd. (Russia) and tap water *ad libitum*. This mouse strain is characterized by moderate incidence of spontaneous tumors and lifespan and is rather frequently used in long-term assays for evaluation of geroprotector potential and carcinogenic safety of drugs [18].

### Test compound

0.005% aqueous solution of BP-C3 was the test compound. The test-compound [1] was supplied by Nobel Ltd. as a powder. It was diluted in distilled water (1 g per 200 ml) with addition of 0.5 ml of 30% ammonia and kept for 15 minutes at 50˚C. Then this basic solution was diluted 200 times with drinking water to the final concentration 0.005%, which was given to animals instead of the drinking water. In preliminary studies good stability of working solution of the test-compound during at least 1 month of storage has been demonstrated. Fresh working solutions were prepared weekly. Daily and average monthly cumulative doses calculated on the basis of regular measurements of body weight and drinking water consumption constituted 10 mg/kg of body weight and 300 mg/kg, respectively.

### Experimental design

One hundred twenty female SHR mice at the age of 3 months were randomized weight-wise into 2 groups, 60 animals each. Animals from one group were used as the intact control, animals from another group were given 0.005% solution of BP-C3 instead of drinking water through their entire lifespan. Animals were monitored daily to assess their condition and to ensure early detection of tumors, mainly, mammary neoplasms, which are typical for this mouse strain [23]. Date of detection, localization and size of tumors were registered in the laboratory logbook. Once a month, all mice were weighted and, simultaneously, the amount of consumed food (g/day/mouse) and water (ml/day/mouse) were measured [44]. Once in every 3 months, daily for 2 weeks, vaginal smears of animals were cytologically examined to assess the estrous function [45]. The age dynamics of the length of estrous cycle and its phases, the rate of estrous cycles of various lengths and fractions of mice with regular and irregular estrous cycles were registered. Rectal temperature was measured at the same time using electric thermometer (TPEM (KMIZ, Russia). Animals were monitored until their natural decease. Date of death, registered for each animal, was used to calculate average lifespan of all animals and the last 10% of surviving animals.

### Pathomorphological examination

All deceased animals were examined post mortem. All tumors, as well as other pathologic changes, major internal organs with suspected tumor infiltration, were excised and fixed in 10% neutral formalin. After routine histological processing the tissues were embedded into paraffin. 4-5 μm thin histologic sections were stained with hematoxylin and eosin and were microscopically examined. Tumors were classified according to International Agency for Research on Cancer recommendations [13].

### Statistics

Results of the experiment were analyzed using variation statistics methods and GraphPad Prism6 software. Average weight and body temperature, food and water consumption were analyzed using TwoWay ANOVA (Holm-Sidak test for multiple comparison). Survival and mortality analyses were performed using Kaplan-Meier log-rang (Mantel-Cox) test. Significance of discrepancies was defined according to the Student t-criterion, Fischer exact method, χ2, non-parametric Wilcoxon-Mann-Whitney test.
